# Diagnostic and Therapeutic Management of Urinary Tract Infections in Catalonia, Spain: Protocol for an Observational Cohort Study

**DOI:** 10.2196/44244

**Published:** 2023-02-22

**Authors:** Ana Moragas Moreno, Silvia Fernández-García, Carl Llor, Dan Ouchi, Ana García-Sangenís, Mònica Monteagudo, Ramon Monfà, Maria Giner-Soriano

**Affiliations:** 1 Institut Català de la Salut, Centre d'Atenció Primària Jaume I Tarragona Spain; 2 Centro de Investigación Biomédica en Red de Enfermedades Infecciosas, Instituto Carlos III Madrid Spain; 3 Universitat Rovira i Virgili Reus Spain; 4 Fundació Institut Universitari per a la Recerca a l'Atenció Primària de Salut Jordi Gol i Gurina Barcelona Spain; 5 Universitat de Girona Girona Spain; 6 Universitat Autònoma de Barcelona Bellaterra (Cerdanyola del Vallès) Spain; 7 Research Unit for General Practice, Department of Public Health University of Southern Denmark Odense Denmark; 8 Spanish Clinical Research Network, Clinical Research Unit Fundació Institut Universitari per a la Recerca a l'Atenció Primària de Salut Jordi Gol i Gurina Barcelona Spain

**Keywords:** urinary tract infection, primary health care, anti-bacterial agents, diagnosis, drug resistance, electronic health records

## Abstract

**Background:**

Antibiotic resistance is an individual and public health problem; multidrug-resistant infections could cause an estimated 10 million deaths worldwide by 2050. Unnecessary use of antimicrobials is the most important cause of resistance generation in the community, and an estimated 80% of antimicrobials are prescribed in primary health care, frequently for urinary tract infections (UTIs).

**Objective:**

This paper presents the protocol for the first phase of the Urinary Tract Infections in Catalonia (Infeccions del tracte urinari a Catalunya) project. We aim to examine the epidemiology of the different types of UTIs in Catalonia (an autonomous community in Spain) and their diagnostic and therapeutic management by health professionals. Furthermore, we aim to evaluate the correlation between types and total consumption of antibiotics for recurrent UTIs in 2 cohorts of women with the presence and severity of infectious complications of urological origin, especially pyelonephritis and sepsis, and 2 potentially serious infections: pneumonia and COVID-19.

**Methods:**

The study is a population-based observational cohort study including adults with a diagnosis of UTI registered in the Information System for the Development of Research in Primary Care (in Catalan: Sistema d’informació per al desenvolupament de la investigació en atenció primària), the Minimum Basic Data Sets of Hospital Discharges and Emergency Departments (in Catalan: Conjunt mínim bàsic de dades a l’hospitalització d'aguts i d’atenció urgent), and data from the Hospital Dispensing Medicines Register (in Catalan: Medicació hospitalària de dispensació ambulatòria) of Catalonia from the period between 2012 and 2021. We will evaluate the variables obtained from the databases to analyze the proportion of different types of UTIs, the percentage of adequate antibiotic treatments prescribed or received for recurrent UTIs according to the national guidelines, and the proportion of UTIs with complications.

**Results:**

We expect to describe the epidemiology of UTIs in Catalonia from 2012 to 2021, as well as describe the diagnostic and therapeutic management of UTIs by health professionals.

**Conclusions:**

We expect to find a high percentage of UTI cases with inadequate management according to the national guidelines, considering that on many occasions UTIs are treated with second- or third-line antibiotic therapies with a preference for the longest regimens. Furthermore, the use of antibiotic suppressive therapies, or prophylaxis, in recurrent UTIs will likely be highly variable. Moreover, we aim to determine whether women with recurrent UTIs treated with antibiotic suppressive therapies have a higher incidence and severity of potentially serious future infections, with special attention to acute pyelonephritis, urosepsis, COVID-19, and pneumonia, compared to women who receive antibiotic treatment after they present with a UTI. This is an observational study of data from administrative databases that will not allow causality analysis. The limitations of the study will be handled according to the appropriate statistical methods.

**Trial Registration:**

European Union Electronic Register of Post-Authorisation Studies EUPAS49724; https://www.encepp.eu/encepp/viewResource.htm?id=49725

**International Registered Report Identifier (IRRID):**

DERR1-10.2196/44244

## Introduction

It is estimated that 10 million people could die worldwide by 2050 from infections caused by multidrug-resistant pathogens if urgent measures are not taken to combat the problem of antimicrobial resistance, mainly among gram-negative bacteria and especially enterobacteria [1]. Antibiotic resistance is an individual health and public health problem; increasing community resistance can prolong the infectious process, increase the risk of infecting other individuals, and in the long term, lead to increased costs. Inappropriate use of antibiotics increases the frequency of related and sometimes serious side effects and causes increased mortality [2,3]. Unnecessary use of antimicrobials is the most important cause of resistance generation in the community, and it is estimated that 80% of antimicrobials are prescribed in primary health care (PHC) [4].

Urinary tract infections (UTIs) are a common infection in PHC, and most patients are treated with antibiotics [5-7]. In Spain, UTIs represent the second most frequent cause of antibiotic prescriptions [8]. Almost 60% of women experience at least one episode during their lifetime [9,10]. Antibiotic treatment of uncomplicated UTIs is, in most cases, empirical, and the selection of the antimicrobial is made according to the most frequently involved pathogen and local resistance. Several studies have suggested that up to 50% of antibiotic prescriptions are inappropriate [11-13]. There is a tendency to use broad-spectrum antibiotics, which do not usually provide substantial improvements in efficacy over narrower-spectrum antibiotics and instead favor increased resistance [4].

Approximately 80% of cases of uncomplicated UTI are caused by *Escherichia coli*, so empirical treatment should cover this pathogen [14-16]. The resistance of uropathogens to classical antibiotics has increased significantly in recent years in Spain [17]. Various studies in Spain have described resistant *E coli* infections and shown that a high percentage are resistant to penicillins. A study published in 2017 that was conducted in our setting among patients with community-acquired pyelonephritis showed that 23% were resistant to ciprofloxacin, while 1% were resistant to fosfomycin [18]. In the program of surveillance of nosocomial infections in the hospitals of Catalonia (Vigilància de les infeccions nosocomials als hospitals de Catalunya [VINCat; all non-English abbreviations are in Catalan unless otherwise noted]), a local database from Catalonia (an autonomous community in Spain) that is part of the Antimicrobial Optimization Program (Programa d’optimització de l’ús d’antimicrobians [PROA]), it was shown that in 2020 *E coli* had a high sensitivity to fosfomycin (96.6%) and nitrofurantoin (98.5%); by contrast, its sensitivity to the amoxicillin/clavulanic acid combination, cotrimoxazole, and quinolones was much lower (75%, 74%, and 72%, respectively) [19]. Resistance of enterobacteria to third-generation cephalosporins, mediated by the production of extended-spectrum β-lactamases, is a growing problem in *E coli* and *Klebsiella pneumoniae* strains. Data from the VINCat PROA 2020 report show that the proportions of β-lactamase-producing *E coli* and *K pneumoniae* urinary tract infections in the community in Catalonia were 9% and 11%, respectively [19]. UTIs caused by resistant microorganisms are associated with longer symptom duration than infections caused by sensitive strains, and treatment is more likely to fail [20].

According to the recommendations of the Infectious Diseases Society of America, empirical antibiotherapy should not be used when resistance rates exceed 20% for all strains [21]. This means that the use of combined amoxicillin/clavulanic acid, as well as quinolones and cotrimoxazole, should no longer be recommended for empirical treatment of UTIs in our country. Current guidelines in PHC recommend prescribing a single dose of 3 grams of fosfomycin trometamol or nitrofurantoin for 5 to 7 days [22,23]. The rationale for this strategy is to specifically target the etiologic agents causing acute cystitis based on knowledge of the local resistance patterns to antimicrobials of the most frequent uropathogens [24]. In recent years, the use of fosfomycin as the therapy of choice for these infections has increased significantly in Spain. However, more than half of all physicians prefer the use of short-course therapies to single doses [25]. One of the reasons mentioned by professionals for not giving first-line treatments is to prevent complications, the most frequent being acute pyelonephritis; however, there is currently no predictive scale available to determine the risk that a UTI will worsen to pyelonephritis. Only 26% of antibiotic prescriptions for uncomplicated UTI were for fosfomycin trometamol (3-gram single dose) or nitrofurantoin in primary care management in Barcelona in the entire year 2020 (unpublished data).

Another dilemma faced by practitioners is the recommendation to give suppressive antibiotic treatment lasting more than 6 months to women who have repeated UTIs (defined as more than 2 UTIs in the last 6 months or more than 3 in the last year) [26]. However, this practice is not exempt from potential risks. In a recent article, a clear correlation was reported between a history of antibiotic use in the previous 2 years and the severity of COVID-19 (with severity defined as a variable combining death, hospitalization, and the presence of pneumonia), based on data from the Information System for the Development of Research in Primary Care (Sistema d’informació per al desenvolupament de la investigació en atenció primària [SIDIAP]) [27]. This correlation was very strong when antibiotics were taken in the previous 2 months, when there were more than 5 antibiotic regimens in the last 2 years, and when antibiotics were used that are critical and should be reserved for special cases, such as cephalosporins and quinolones. In addition, prudent use of antibiotics is necessary due to the increase in antibiotic resistance [3,28].

Urine culture requests are not always made according to guidelines. As early as 2000, it was documented that 44% of requests were made for cases of asymptomatic bacteriuria, a condition for which they should not be ordered and there should be no treatment [29]. In 2014, 92% of urine cultures that were ordered for a second time for patients with a UTI were not necessary [30]. In 2019, a range of 21% to 42% of requests for urine cultures were for uncomplicated UTIs in Barcelona city primary care management (in Spanish: gerencia *de atención primaria de Barcelona ciudad*), depending on the primary care center (unpublished data). The ordering of urine cultures for women with cystitis is only recommended in cases of (1) uncomplicated cystitis that, despite adequate antibiotic treatment, remains symptomatic (posttreatment urine cultures are used), (2) recurrent cystitis (pretreatment urine cultures are used), and (3) cystitis in pregnancy (both pretreatment and posttreatment urine cultures are used) [14]. The use of these tests when they are not necessary leads to antibiotic overprescription; moreover, since in many cases they are requested for cases of bacteriuria in elderly people who have been treated with antibiotics many times, the results of antibiograms recommend the prescription of second-line antibiotics for uropathogens that are often multidrug-resistant.

For all the above reasons, we propose the current protocol for the Urinary Tract Infections in Catalonia (Infeccions del tracte urinari a Catalunya [ITUCAT]) study, consisting of 4 work packages (WPs) that will be implemented successively in 3 phases, to evaluate different objectives related to UTIs in the adult population in Catalonia and investigate specific aspects of this condition in this population. The first phase of the project, which is presented in this paper, includes the first 2 WPs. WP 1 will evaluate the management of UTIs in Catalonia, and WP 2 aims to evaluate the correlation of antibiotic use for repeated UTIs with the presence of infectious complications in adult women. Subsequently, we will carry out the second phase with the data obtained from the first phase. At this stage, the aim is to create a scale to predict acute pyelonephritis in patients with UTI by evaluating clinical variables; this will be WP 3. Finally, in the third phase, we will perform WP 4, which aims to determine the degree of inadequacy in requests for urine cultures based on clinical practice guidelines and to establish the basis for a training intervention.

The study described in this paper corresponds to the first phase, which includes WP 1 and WP 2.

## Methods

### Study Design and Population

This is a population-based observational cohort study. The inclusion period was from January 1, 2012, to December 31, 2021. The study population was patients aged ≥18 years with a diagnosis of UTI registered in SIDIAP during the study period. UTI diagnoses included the International Statistical Classification of Diseases and Related Health Problems, 10th Revision (ICD-10) codes (Table 1).

**Table 1:** Health problems included in the study and their International Statistical Classification of Diseases and Related Health Problems, 10th Revision codes.

ICD-10 codes	Health problems
N10	Acute tubule-interstitial nephritis
N30	Cystitis
N30.0	Acute cystitis
N30.1	Interstitial cystitis (chronic)
N30.2	Other chronic cystitis
N30.3	Trigonitis
N30.8	Other cystitis
N30.9	Cystitis, unspecified
N34	Urethritis and urethral syndrome
N34.1	Nonspecific urethritis
N34.3	Urethral syndrome, unspecified
N39.0	Urinary tract infection, site not specified
N41	Inflammatory diseases of prostate
N41.0	Acute prostatitis
N41.1	Chronic prostatitis
N41.3	Prostatocystitis
N45	Orchitis and epididymitis
N45.0	Orchitis, epididymitis, and epididymo-orchitis with abscess
N45.9	Orchitis, epididymitis, and epididymo-orchitis without abscess

### Data Collection and Data Sources

The data needed to carry out the project will be obtained from the SIDIAP database, the Minimum Basic Data Sets (Conjunt mínim bàsic de dades [CMBD]) of Hospital Discharges and Emergency Departments (Conjunt mínim bàsic de dades a d’hospitalizació d’aguts [CMBD-HA] and Conjunt mínim bàsic de dades d’atenció urgent [CMBD-UR], respectively) registries, and data from the Hospital Dispensing Medicines Register (Medicació hospitalària de dispensació ambulatòria [MHDA]).

The SIDIAP contains pseudonymized clinical information from the Electronic Health Records in Primary Care (Estació clínica d’atenció primària) program [31], which is the electronic health records program for PHC of the Catalan Health Institute (Institut català de la salut [ICS]) in Catalonia. The ICS manages 279 PHC centers, covering a population of 5.8 million people (approximately 80% of the Catalan population). Among this adult population, information is available for more than 3384 PHC medical staff members.

The information recorded in SIDIAP contains sociodemographic data; health conditions, coded by ICD-10 [32]; clinical parameters; tobacco and alcohol use; diagnostic procedures; PHC laboratory test results; specialists’ referrals; and prescriptions of PHC medical staff, with the corresponding pharmacy billing data, registered as anatomical, therapeutic, chemical (ATC) classification system codes [33]. Several reports have shown that SIDIAP data are useful for epidemiological research [34,35]. SIDIAP is listed under the European Network of Centres for Pharmacoepidemiology and Pharmacovigilance resources database [36].

The CMBD is a population-based registry that collects information on pathologies treated in the health centers of Catalonia [37] and includes ICD-10 codes [32]. This registry contains information provided by all Catalan health care centers on health care activity and morbidity. The CMBD-HA contains information on acute hospitalizations, with reasons and dates for hospital admission. The CMBD-UR reports activity in emergency departments.

The MHDA is a registry containing information on specific drugs that are dispensed in hospitals and reimbursed by the Catalan health system [38,39].

### Sample Size

A feasibility count was requested from the SIDIAP database for the years 2012 and 2021. It was estimated that approximately 2.5 million UTIs were registered in the Catalan population during the study period.

### Variables

Variables included in WP 1 and WP 2 include sociodemographic information; clinical variables and health conditions, with ICD-10 codes; tobacco and alcohol use; PHC laboratory test results; vaccination status (for influenza, pneumococcus, and COVID-19); prescriptions, with their corresponding pharmacy invoice data registered as ATC codes [33] in addition to prescriptions for MHDA drugs [38,39]; dates of sickness leaves, visits, and referrals (to second- and third-level centers); sexual and reproductive health care; CMBD-HA hospital information; and CMBD-UR emergency department information (Multimedia Appendix 1).

### Study End Points

#### Primary End Points

The primary end points of the first phase for WP 1 are the proportion of different types of UTI (uncomplicated UTIs, recurrent UTIs, and pyelonephritis), the percentage of first-line antibiotics used for different types of UTI according to the national guidelines [40,41], and the percentage of inappropriate prescriptions, with the type of antibiotic of these prescriptions. The primary end points for WP 2 are the percentage hospitalization rate and the proportion of UTIs with complications (ie, pyelonephritis, sepsis, pneumonia, and COVID-19). COVID-19 infection will be determined by ICD-10 codes, tests (ie, polymerase chain reaction tests, rapid antigen tests, and others), or both.

#### Secondary End Points

The secondary study endpoints are (1) types of UTI in women aged ≥18 years with the percentage of recurrent UTIs (defined as more than 2 UTIs in the last 6 months or more than 3 episodes in the last year); (2) types of UTI in men aged ≥18 years; (3) the percentage of urine cultures and laboratory tests ordered for the different types of UTI in both men and women; (4) the percentages of different types of antibiotics used in patients with UTIs, as well as dosage and duration of treatment; (5) the percentage of women with recurrent UTIs who were treated with suppressive therapy (ie, antibiotic treatment 3 times a week for 6 months or more) and the duration of the therapy; (6) the percentage of infectious complications, especially acute pyelonephritis and sepsis, in the 2 cohorts (ie, women with recurrent UTIs with suppressive therapy and women with recurrent UTIs without suppressive therapy but with UTIs treated punctually; (7) the percentage of pneumonia and COVID-19 infection in the 2 cohorts (ie, women with multiple antibiotic regimens for UTIs or suppressive therapy and women with UTIs treated punctually); and (8) the frequency of clinical variables of people affected by UTI who progress to acute pyelonephritis.

### Statistical Analysis

Demographic and baseline characteristics of the participants will be reported as frequencies and percentages for categorical variables and means and standard deviation or median and interquartile range for continuous variables, as appropriate.

For WP 1, descriptive statistics will be reported for the results. For WP 2, a severity variable will be constructed that will include mortality and hospitalization, in addition to the presence of pneumonia in cases of COVID-19. Quantitative variables will be described as means and standard deviation, while categorical variables will be described as the proportion of exposed and unexposed individuals. Univariate tests will include the Student *t* test and the chi-square test, as appropriate. For the primary outcome, marginal structural models will be fitted to estimate causal effects by correcting for confounders. Inverse probability weights will be estimated as a function of propensity score using sociodemographic variables and clinical variables. The inverse probability weights will be used in the marginal structural model to estimate the risk ratio and confidence intervals for the prevalence of each outcome among individuals exposed to antibiotics versus those not exposed to antimicrobials. During the assessment of the correlation of prior antibiotic exposure with the different outcomes, patients will only be counted once and assigned to the worst outcome (with the severity of COVID-19 ranked in decreasing order as death, hospitalization, and pneumonia). The Wald test will be used to determine whether the adjusted risk ratios are significantly different from zero at a significance level of 5%.

### Ethical Aspects and Data Confidentiality

The present study follows national and international regulations, including the Declaration of Helsinki Ethical Principles for Medical Research Involving Human Subjects, Good Research Practice principles and guidelines, and the *Real Decreto 957/2020, de 3 de noviembre*, which regulates observational studies of medicines for human use. The study protocol was approved by the Institut Universitari d’Investigació en Atenció Primària Jordi Gol Clinical Research Ethics Committee, the reference institution for research in primary health care of the ICS, on September 27, 2022. In accord with Spanish legislation on confidentiality and data protection (*Ley Orgánica 3/2018, de 6 de diciembre de 2018, de Protección de Datos Personales y garantía de los derechos digitales*), data included in SIDIAP are always pseudoanonymized. Thus, it is not necessary to obtain informed consent from the participants.

## Results

We expect to describe the epidemiology of UTIs in Catalonia during the period from 2012 to 2021, including the characteristics of the population, diagnoses of UTI, the use of urine cultures or laboratory tests, and different antibiotics prescribed according to treatment guidelines for dose and duration. Furthermore, we will report the degree of adequacy of the prescribed treatments and the clinical evolution of the patients (ie, the rates of clinical resolution, recurrence, hospitalization, and complications, such as acute pyelonephritis, urosepsis, pneumonia, COVID-19, and death).

The first phase of the ITUCAT project is expected to be completed during the second half of 2023. Phase 2 will start after the data from phase 1 are obtained and used to create the predictive scale for acute pyelonephritis in patients with UTI; phase 2 is expected to be completed in 2024. Phase 3 will be carried out during the second half of 2023 and during 2024 to evaluate the degree of inadequacy in requests for urine cultures according to the clinical practice guidelines and establish the basis for a training intervention.

The results obtained will be presented according to the Reporting of Studies Conducted Using Observational Routinely Collected Health Data Statement for Pharmacoepidemiology (RECORD-PE) recommendations [42].

## Discussion

We expect to find inadequate management in a high percentage of UTI cases, considering that on many occasions patients are treated with second- or third-line antibiotic therapies with a preference for the longest regimens. Likewise, the use of suppressive antibiotic therapy as a prophylaxis in cases of recurrent UTI is expected to vary in our study.

In addition, we aim to determine whether women with recurrent UTIs treated with antibiotic suppressive therapy have a higher incidence and severity of potentially serious future infections, with special attention to acute pyelonephritis, urosepsis, COVID-19, and pneumonia, in comparison to women who are treated with antibiotics only when they present with a UTI.

All the information obtained in this first phase will allow us to continue with the following WPs of the project. Furthermore, if we find the inappropriate use of antibiotics, we will work on the creation of proposals to promote the correct treatment of UTIs in our environment according to clinical guidelines [40,41]. In this way, we will promote responsible management of UTIs in PHC among health care personnel and patients. In addition, our study will provide valuable information for health care policy makers and national programs to optimize the use of antimicrobials.

Because this is an observational study based on data from administrative databases, it will not allow causality analysis. However, the results will be derived from health care carried out under conditions of routine clinical practice with data from electronic primary-care registries, including the SIDIAP, CMBD-HA, and CMBD-UR databases, which have proven to be valid and representative of the population in numerous previous studies [31,34,35]. Some limitations of these studies arose from underreporting of some variables and diagnoses that we will try to avoid with the use of the different databases. Another limitation of the databases is the lack of links between diagnoses and treatments. In order to deal with bias from this specific limitation, we plan to use proxy values available in the databases, such as dates of prescriptions and dates of diagnoses. An inherent limitation of observational studies is the presence of unmeasured confounding variables. A complete adjustment for all possible confounders would require detailed information on clinical parameters, lifestyles, socioeconomic conditions, use of over-the-counter medications, and personal circumstances, which is not routinely present in electronic health records, causing residual confounding bias. Nevertheless, this bias would affect all included populations equally. To minimize confounding, we will conduct sensitivity analyses and use external adjustments or specific statistical methods when appropriate [43]. We will also take this aspect into account in the scientific discussion of the results, as it is an inherent limitation of studies conducted with electronic health records.

## Appendix

Multimedia Appendix 1 Description of the first phase variables.

## Abbreviations

ATC: anatomical, therapeutic, chemical
CMBD: Conjunt mínim bàsic de dades (Minimum Basic Data Set)
CMBD-HA: Conjunt mínim bàsic de dades a d’hospitalizació d’aguts (Minimum Data Set of Diagnoses at Hospital Discharge)
CMBD-UR: Conjunt mínim bàsic de dades d’atenció urgent (Minimum Data Set of Diagnoses at Emergency Departments)
ICD-10: International Classification of Diseases, 10th Revision
ICS: Institut català de la salut (Catalan Health Institute)
MHDA: Medicació hospitalària de dispensació ambulatòria (Hospital Dispensing Medicines Register)
SIDIAP: Sistema d’informació per al desenvolupament de la investigació en atenció primària (Information System for the Development of Research in Primary Care)
PHC: primary health care
UTI: urinary tract infection
WP: work package

## Data Availability

The data sets used and analyzed are available from the corresponding author on reasonable request.
