# Supplementary material for: Diagnostic and Therapeutic Management of Urinary Tract Infections in Catalonia, Spain: Protocol for an Observational Cohort Study
Source: JMIR Res Protoc. 2023 Feb 22;12:e44244. doi: 10.2196/44244 (PMC9996422; doi:10.2196/44244)
Supplement: Multimedia Appendix 1 [file resprot_v12i1e44244_app1.pdf]

MULTIMEDIA APPENDIX

Description of the first phase variables.

GENERAL PATIENT INFORMATION
Date of birth without day for confidentiality reasons
Sex
Nationality
Patient status on date of last data update (active, transferred, or deceased)
Primary Care Center
Professional
Complexity indicator Clinical Risk Groups
Institutionalized
Frailty status of the patient
Morbidity Adjusted Groups (GMA) complexity

CLINICAL VARIABLES
International classification of diseases (ICD) 10 code
Smoking

SOCIOECONOMIC INDICATORS
Mortality in small Spanish areas and economic and environmental inequalities (MEDEA) quintil
Rurality
Medea center quintile

HEALTH PROBLEMS
International classification of diseases (ICD) 10 code
Charlson Comorbidity Index (CCI)

LABORATORY AND SEROLOGY
Urine biochemistry
Blood biochemistry
Exudate biochemistry
Cultures
Haematology

VACCINES
Influenza
Pneumococcus
COVID-19

VISITS

Service of the visit

Type of visit (eConsultation, on-site visit, home)

Referrals

SEXUAL AND REPRODUCTIVE HEALTH CARE

Date of last menstruation

End date of pregnancy

Circumstance of end of pregnancy

Risk of the pregnancy

Mother’s history of pregnancies

SICK LEAVE FROM WORK

International classification of diseases (ICD) 10 code of the health problem causing sick leave

Date of sick leave and discharge

COVID-19 VARIABLES

Covid-test

COVID-Monitoring form (clinical variables)

HOSPITAL DATA

Minimum basic data sets of diagnoses at hospital discharge -CMBD-AH-

Minimum basic data sets of diagnoses at emergency department – CMBD-UR-

INVOICED AND PRESCRIBED DRUGS (ATC code)

A10	Drugs used in diabetes mellitus
C02	Antihypertensive drugs
C03	Diuretics
C04	Pheripheral vasodilators
C07	Beta-blockers
C08	Calcium channel blockers
C09	Agents acting on the renin-angiotensin system
G01	Gynecological antiinfectives and antiseptics
G02B, G02CC, G02CS	Other gynecologicals
G03C, G03D, G03F, G03XX	Sex hormones and modulators of the genital system
G04C, G04BC, B04BD	Urologicals
H02	Corticosteroids for systemic use
J01	Antiinfectives for systemic use
L01	Antineoplastic agents
L02	Endocrine therapy
L04A	Immunosuppressants
M04AC	Preparations with no effect on uric acid metabolism
R03BA	Glucocorticoids
R03DC, R03DX	Other systemic drugs for obstructive airway disease
P01BA02	Hydroxychloroquine

HOSPITAL DISPENSING MEDICINES REGISTER -MHDA- (ATC code)

A07EC03	Olsalazine
D11AH05, D11AH07	Agents for dermatitis, excluding corticosteroids
G02BA03, G02CB01	Other gynecologicals
G03CA01, G03CA57, G03DA02, G03FB06	Sex hormones and modulators of the genital system
G04BC	Urinary concrement solvents
H02AB08, H02BA09	Glucocorticoids
J04BA02	Dapsone
L01	Antineoplastic agents
L02AA01, L02BB04, L02BB05, L02BB06, L02BX03, L02BX3	Endocrine therapy
L04A	Immunosuppressants
R03DX10	Benralizumab
L04A	Immunosuppressants
M04AC	Preparations with no effect on uric acid metabolism
R03BA	Glucocorticoids
R03DC, R03DX	Other systemic drugs for obstructive airway disease
P01BA02	Hydroxychloroquine
